# Medicinal plants for treatment of diarrhoeal diseases among under-five children: experience from traditional healers in North-eastern Tanzania

**DOI:** 10.1186/s12906-023-04216-0

**Published:** 2023-10-25

**Authors:** Edwin Liheluka, Nyasiro Sophia Gibore, John P. A. Lusingu, Samwel Gesase, Daniel T. R. Minja, Maike Lamshöft, Denise Dekker, Theodora Bali

**Affiliations:** 1 Department of Public Health and Community Nursing, University of Dodoma, Dodoma, Tanzania; 2 National Institute for Medical Research (NIMR), Tanga Centre, Tanga, Tanzania; 3 Bernhard Nocht Institute for Tropical Medicine, BNITM, Hamburg, Germany; 4 German Center for Infection Research, DZIF, Braunschweig, Germany; 5 University Hospital Hamburg-Eppendorf, Hamburg, Germany; 6 Department of Educational Psychology and Curriculum Studies, University of Dodoma, Dodoma, Tanzania

**Keywords:** Medicinal plants, Herbal medicines, Traditional healers, Under-five children, North-eastern Tanzania, and diarrhoeal diseases

## Abstract

**Background:**

Right through history, humans have relied heavily on plants for sustenance and the healing of different ailments. One of the long-standing traditions that communities have inherited from earlier generations is the use of herbal medicines for the treatment of paediatric ailments, including diarrhoea. This study showcased medicinal plants used by traditional healers for the treatment of diarrhoeal diseases among under-five children in North-eastern Tanzania.

**Methods and design:**

A qualitative research approach and a narrative research design were employed. The research was carried out in the districts of Korogwe and Handeni in North-eastern Tanzania, with 52 in-depth interviews performed with participants (traditional healers). Purposive sampling method was used to select participants, and a thematic analysis framework was used to analyze the data.

**Results:**

Study results indicate that traditional healers had enormous insights and were well informed about medicinal plants that were perceived to be efficacious in treating diarrhoeal diseases among under-five children. A total of 54 medicinal plants were reported by the participants to be effective in healing diarrhoeal diseases among under-five children. However, out of 54 medicinal plants, 15 were predominantly disclosed by the majority of participants. Those medicinal plants include *Psidium guajava, Rhus natalensis, Ozoroa insignis, Tamarindus indica, Ocimum suave, Combretum molle, Zanha africana, Solanum incanum*, and *Ximenia americana.* Other medicinal plants mentioned by most participants include, *Ochna holstii, Elaeodendron schlechterianum, Albizia anthelmintica, Commiphora pteleifolia, Salacia stuhlmanniana*, and *Zenkerella grotei.*

**Conclusion:**

All traditional healers seemed to have a clear understanding regarding the medicinal plants that were used to treat diarrhoeal diseases among under-five children. The participants acknowledged to treating under-five children with diarrhoeal diseases using herbal medications on multiple occasions. The findings of this study should inspire more in-depth botanical research to determine whether the medicinal plants reported in this study have anti-diarrhoeal properties.

## Background

Products derived from specific medicinal plants, such as leaves, barks, roots, seeds, and fruits, are said to be a great source of medicines for both humans and animals and have been used since ancient times to solve a variety of health ailments [1]. To date, there are communities that are believed to have not yet been reached by modern health care facilities and medications, hence herbal medicines continue to be the sole viable treatment option for individuals in those settings [2]. In non-industrialized countries, herbal medicines have been in great demand given that modern medicines are often perceived as inaccessible, expensive, and unsuitable [3]. Studies emphasize the need for more research and conservation strategies on herbal medicines since there has been concern about medicinal plants disappearing in the near future, mostly due to climate change, environmental destruction, and overharvesting of these products [4]. Over 20% of wild medicinal plant species are thought to have nearly completely disappeared from their original habitats on a global scale [4].

Various medicinal plants are believed to be efficacious in healing a range of diseases, although there is limited scientific data to provide authentic evidence about the safety and effectiveness of most of the herbal medicines [5, 6]. Having realized this challenge and due to the essential role that herbal medicines play in responding to the health problems of the majority of people across the world, the World Health Organization (WHO) is presently working on accomplishing the “Traditional Medicine Strategy 2014–2023”. The deliberate goal of this strategy is to draft principles and scientific references that will help its members deliver tenable and worthwhile herbal medicine services [7].

In Tanzania, the use of herbal medicines is embedded within the culture of many communities [8, 9]. It is estimated that about 60% of Tanzanians receive medical care from Traditional Healers (THs) prior to their initial contact with formal healthcare settings [10]. A study from Eastern Tanzania showed that over 70% of caretakers who participated in the study reported using herbal medicines to manage diarrhoeal ailments in their under-five children [11]. Likewise, a study conducted in North-eastern Tanzania revealed that about 70% of study participants preferred the use of herbal over modern medicines in treating various ailments, including diarrhoea [9]. Studies conducted in other settings suggest that the medicinal plants that are routinely used to treat diarrhoeal diseases come from the family of *“*Anacardiaceae, Lamiaceae, Asteraceae, Clusiaceae, Myrtaceae, Mimosaceae, Rubiaceae Fabaceae and Malvaceae*”* [9, 12–14]. Despite their claimed efficacy for diverse functions, including defense and shelter against diarrhoeal diseases among under-five children, some herbal medicines have been reported to have adverse effects such as nausea, lassitude, vomiting and stomachache [11]. Therefore, the use of these herbs in treating diarrhoeal diseases requires awareness, and thus the need for service providers to register themselves and their herbal products becomes mandatory as the Tanzanian government demands [15].

Diarrhoea is a poverty-associated disease that occurs overwhelmingly frequently in the poorest parts of the world with deprived access to standard health care [16]. The disease is estimated to kill 1,400 young children every day in the world [17]. Significant numbers of diarrhoeal deaths occur among children aged less than two years who reside in resource-constrained settings [17]. In Tanzania, despite various interventions in managing diarrhoeal diseases, such as the introduction of the rotavirus vaccine in 2013 [18], the overall prevalence of diarrhoea in the country among under-five children still remains high, at about 25% in 2015 [19].

Herbal medicines have been demonstrated in studies to be among the interventions that are commonly used to address diarrhoeal illnesses in under-five children [11, 20]. However, their contributions in curbing the ailments are not well known [6, 12, 20, 21]. According to statistics, the Tanga Region is experiencing a significant public health issue with diarrhoea in under-five children. For instance, the prevalence of diarrhoea among under-five children in Tanga Region was 53% in 2017, 56% in 2018, 60% in 2019, and 59% in 2020 [22]. While this has been happening, the use of herbal medicines to treat diarrhoea in under-five children has been fully embraced by the relevant community [9, 23, 24]. Thus, the purpose of this study was to identify the medicinal plants that were most used to treat diarrhoea among under-five children from the perspectives of THs in Korogwe and Handeni districts, Tanga Region, North-eastern Tanzania. The knowledge of these medicinal plants may possibly provide an opportunity for more scientific research to be done in order to establish their efficacy and safety, unlike now, where most of the information is only word of mouth from THs, that herbal medicines are safe and effective.

In addition, understanding the medicinal plants that are used to treat paediatric diarrhoea might offer a foundation of comprehension for establishing cutting-edge diarrhoea-related health interventions. Furthermore, documenting kinds of medicinal plants relevant for treatment of diarrhoeal diseases among under-five children, may also serve as local knowledge source of medicinal plants for future generations. Last but not the least the information can be harnessed to prepare a herbarium that can assist the general public and country policies on valuable herbal medicines of crucial values to the Tanzanian society and beyond.

## Methods

### Study setting

The present study was carried out in Korogwe and Handeni districts, Tanga Region in North-eastern Tanzania from June 2022 to March 2023. The two districts had been earmarked for this research since the majority of the population noticeably embodied the practices of utilizing herbal medicines for management of diverse illnesses, including diarrhoeal diseases, among under-five children [9, 24]. Among the factors cited as contributing to the widespread use of herbal medicines in Tanga Region included the abundance of medicinal plants that were found within the region and the existence of a large numbers of THs whose ratio to population ranged between 1:343 in urban areas and 1:146 in rural areas [24].

Korogwe District has an area of 3,756 square kilometers [25] and an approximate population of 359,421 [26]. Topographically, the district is mainly occupied by the scenic Usambara highlands as well as the Pangani and Lwengera River valleys [25]. Handeni District has an area of 6,453 square kilometers [27] and a population of 493,321 [26]. The district is divided into two main zones namely, the upper zone and the plain zone. The upper zone is made up of scattered mountain ridges and peaks and has taken up about 75% (4,839.75 km^2^) of the entire area of ​​Handeni District, while the plain zone has an elevation between 200 and 400 m from the sea level and it covers almost 25% (1,613.25 km^2^) of the entire area of ​​the district [27].

### Study approach and design

In the present study, a qualitative research approach and a narrative research design were used. The approach and design were chosen based on the study aims, which were mostly exploratory [28]. The study aimed at getting insights related to medicinal plants for the treatment of diarrhoeal diseases among under-five children from the perspectives of THs working in Korogwe and Handeni districts.

### Study population, inclusions, and exclusions criteria

The study population consisted of inhabitants from Korogwe and Handeni districts, with the target population consisting of THs who were working within the two districts. The THs were chosen since they were mostly responsible for managing diarrhoeal cases by using herbal medicines. Experienced THs who treat under-five children in Korogwe and Handeni districts were included in the study. Traditional healers who could not participate in the study due to illness and those who were absent on the day of data collection were excluded from the study.

### Sampling technique of study participants

Purposive sampling technique was used to select participants. The technique was chosen since it is a technique that is broadly preferred in qualitative studies as it allows the researcher to select and include participants in a specific study who are informed and experienced on the topic of inquiry [29].

### Data collection methods and tools

In order to facilitate data collection for this study, an in-depth interview (IDI) was used. The method was selected for the reason that it is the most practical for collecting qualitative data in health research [29], since it allows the researcher to gain greater thoughtfulness related to the subject matter (in this case, medicinal plants). In terms of data collection tools, an IDI guide with open-ended questions was employed to aid data collection. The guides developed were related to the study objectives and were prepared in English before being translated into Kiswahili.

### Procedures for conducting IDIs meetings

#### Sensitization meetings

Prior to the initiation of the formal IDI interviews with THs, the Principal Investigator (PI) and research assistants held four meetings with District Medical Officers (DMOs) and Town Medical Officers (TMOs) of the four councils; two meetings were for Korogwe and two for Handeni districts. The aim of these meetings was to raise awareness and give them detailed information about the study as key stakeholders in the health sector within the two districts. During these meetings, study objectives, settings, procedures, benefits, risks, and study duration were described. Following the discussions with important health stakeholders, the team organized two more meetings with district TH coordinators and local TH leaders from all four councils, with the same goal in mind: to present them with detailed information about the study. These meetings were critical to the success of the present study.

### Selection of participants

The present study purposively recruited 52 THs from each of the 10 divisions found in Korogwe and Handeni districts. The TH coordinators of Korogwe and Handeni districts were consulted to select a minimum of four and a maximum of six THs from each division to participate in the present study. Traditional healer coordinators managed to select five THs from each of the eight divisions in Korogwe and Handeni districts. Two divisions, Mombo in Korogwe District and Mkumburu in Handeni District, provided six THs since they were the divisions with the highest number of THs, which made the number, reach 52.  As explained above in the inclusion criteria, THs were selected based on their experiences and reputations in treating children in Korogwe and Handeni districts.

### Conducting IDI meetings

After participants had been selected, the PI located the participants in the areas where they live one week before the interview date and informed them of their selection to participate in the study. The PI took time to enlighten each participant about the study objectives and the relevance of their participation in the study. Similarly, the PI obtained contact details for each participant, such as mobile phone number, village name, and sub-village name; the aim was to facilitate communication before and after the interview.

The study PI, with the help of a research assistants, conducted all 52 IDIs. The interview process started once the participant had consented to participate in the study and had signed and dated the Informed Consent Form (ICF). Information was recorded by one research assistant by taking notes, and after the participant agreed, the digital audio-visual recorder was used. The research assistant was responsible for recording the interviews using the digital audio recorder under the supervision of the PI. One IDI took approximately 20 to 30 minutes to complete. The semi-structured interview guide with open-ended questions was used, and the questions were asked as per the guide. Only one researcher interviewed an IDI participant at a time, while a research assistant took notes. All the IDI interviews were carried out in a place well-situated to the study participant.

### Data management and analysis

Data analysis was performed using thematic analysis, which is a widely used method for identifying themes in the data [30]. The transcription of the interviews, which were first done in Kiswahili and then translated into English, was the first step in the data analysis process. All the transcripts were then reviewed and re-read to become comfortable with the data. To improve the accuracy of the classification, two qualified moderators independently sorted and examined the data. The analysis of the data was routinely evaluated by the research team that conducted the data collection in order to develop the insights and ensure that the findings accurately reflected the study context. Finally, the analysis was conducted using the six stages of theme analysis recommended by Braun and Clarke: Orientation of the information collected, making preliminary codes, finding potential themes, examining themes, defining and naming themes, and finally producing the report [30] as explained below.

Orientation of the information collected: To understand the abundance of the information in connection to the study objectives, the data collected were familiarized. Potential codes were identified at this stage, and they were eventually used when composing the final report.

Making preliminary codes: Preliminary research objectives-related codes were drafted at this point and organized into a useful category, the emphasis was on creating key points that made it easier to connect participant reports to the study objectives. To increase the reliability of the classification, the data were separately categorized and analyzed by two knowledgeable moderators.

Finding potential themes: After all the data had been formally coded, the investigator organized the various codes into potential research themes and assembled the properly coded data into the designated study themes.

Examining themes: After the data had been organized into potential research themes, the themes were demonstrated to determine if they had sufficient supporting information. After critically reviewing the themes, other themes were modified and combined. Other themes that appeared to lack enough supporting data were removed.

Defining and naming themes: At this stage the themes were scanned, evaluated, and further enhanced in order to make them suitable for the final data analysis after being identified as prospective themes with adequate supporting data. Before generating the report, two skilled moderators went through the analysis process, which was carried out independently step by step, and they both agreed that the themes generated were based on the information gathered from the participants. After then, the report writing procedure started by adhering to the order of the structured themes.

### Trustworthiness

The present study’s trustworthiness is consistent with the credibility, transferability, dependability, and confirmability of the study results, which were achieved after the study team conducted interviews with study participants.

**Credibility**: By being able to execute the proposed scientific research methods, carrying out the member checks model and giving respondents the freedom to participate or not in the study, the findings of this study are trustworthy as recommended by the credibility decree [31].

**Transferability**: In order to ensure the results, which have been obtained from this study, can also be obtained elsewhere if conducted by a different researcher, the present study clearly stated the inclusion and exclusion criteria for participants who took part in this study. This study also described the study setting and the institution (The University of Dodoma) that oversaw the conduct of the study. The present study adhered to the above criteria, which are key in determining the legality of the study findings should other research of this kind be conducted elsewhere by different researchers [30].

**Dependability**: For this to be achieved, the present study implemented what was initially proposed in the study proposal. Moreover, the study managed to sufficiently describe the entire flow of the research conduct from the beginning to the very end until the results were obtained. By being able to wholly describe the study conduct, the findings of this study are dependable, as recommended by the dependability order [31].

**Confirmability**: The present study demonstrated how data were collected after sensitizing the study communities, and the quotes deployed in the study results were exactly extracted from the transcripts, which were personal narratives from study participants involved in IDIs [31].

## Results

### Demographic characteristics of study participants

A total of 52 participants took part in this study, of whom 32 (61.5%) were males and 20 (38.5%) were females, with ages ranging from 29 to 80 years. A high proportion of participants 41 (79%) had primary school education, while 4 (8%) participants had secondary school education, and 7 (13%) participants had not attended school, (Table 1). However, there was a notable sex difference in their levels of education, with slightly more male participants attaining primary and secondary school education levels compared to females. Years of working experience as traditional healers ranging from two years to 61 years.

**Table 1:** Socio-demographic characteristics of study participants

Characteristics	Male (n = 32) 61.5%	Female (n = 20) 38.5%	Total (N = 52) 100%
**Age (years)**			
Younger age	1	0	1
Middle age	11	12	23
Older age	20	8	28
**Career**			
Traditional Healers	32	20	52
**Data collection methods**			
In-depth interviews	32	20	52
**Level of education**			
No formal education	3	4	7
Primary education	25	16	41
Secondary education	4	0	4
**District**			
Korogwe	12	9	21
Handeni	17	14	31
Years of working experience as TH			
1–10	11	14	25
11–20	7	4	11
21>	14	2	16

The findings of this study are presented in the following themes that emerged during analysis: (1) Treating diarrhoeal diseases by using herbal medicines among under-five children, (2) Medicinal plants for the treatment of diarrhoeal diseases among under-five children, and (3) Therapeutic effects of herbal medicines relevant for diarrhoeal diseases treatment among under-five children.

### Treating diarrhoeal diseases by using herbal medicines

All interviewed participants (THs) acknowledged that they have treated under-five children who were suffering from diarrhoeal diseases by using herbal medicines, and to date they still continue to treat them. They claimed that their knowledge of treating various diseases, including diarrhoea, was acquired by being taught and inherited from their parents/guardians, grandparents. Others claimed to have learnt about herbal treatments through spirits, where teachings were coming to them in the form of dreams while sleeping at night. None of the THs had ever attended formal education related to herbal medicines. For instance, participants had this to say:

*“I have treated them a lot”* Participant no 06, Male 51-year-old.

Another participant echoed what was said:

*“Yes, I have treated them a lot”* Participant no 44, Female 50-year-old.

Additionally, another participant disclosed:

*“I have treated my own children”* Participant no 07, Male, 50-year-old.

Regarding the expertise of treating people by using herbs, participants commented:

“*I inherited it from my father and my maternal grandmother”* Participant no 12, Male 66-year-old.

*“I was taught by my late elders”* Participant no 15, Male, 51-year-old.

A few THs were of the view that they had acquired the knowledge of healing through spirits, and when they were asked to clarify more on how the spirits taught them, they could not clearly elucidate:

*“This job is originated from ghosts”* Participant no 14, Female 45-year-old.

*“No one taught me; it came naturally; I was not like this, but I was sick for two years, and they (spirits) ordered me to go into the forest and pick herbs, and then they instructed me to boil them”* Participant no 16, Female 42-year-old.

Participants were of the view that their expertise in treating people by using herbal medicines makes them highly respected in the society, and the majority of people tend to hurry to their services, believing that they will be healed. From their point of view, they believe that caretakers’ faith in them and their ability to treat under-five children with diarrhoea is the main reason why they continue to be appreciated by the wider community. For example, participants declared:

*“It’s the faith that allows us to have access to these patients in the first place. I think they have a lot of faith in us. When a person knows that they believe in herbal medicines that it is enough for them, then going to the hospital rarely happens”* IDI, Participant No 37, Female, 50-year-old.

*“Yes, I have treated young children who were suffering from diarrhoeal diseases in Handeni District by using herbal medicines”* IDI, Participant No 52, Male, 42-year-old.

*“Many times, it is because of faith. I have treated young children who were suffering from diarrhoeal diseases using herbal medicines”* IDI, Participant No 47, Female, 37-year-old.

### Medicinal plants for the treatment of diarrhoeal diseases among under-five children

A total of 54 medicinal plants have been reported as having therapeutic effects for treating diarrhoeal illnesses among under-five children (Table 2). According to the majority of study participants, the roots, barks, and leaves of the specific medicinal plant were the most important plant parts with a high concentration of medication. Table 2 below provides detailed specific information about the plant’s local language name, scientific botanical name, family name, plant parts utilized, and how the herbs were prepared.

**Table 2:** Medicinal Plants for diarrhoea treatment among under-five children

SN	Local language name of the plant	Scientific Botanical Name	Family Name	Parts used	How the medicine is prepared
1	Mpera - (Swahili)	*Psidium guajava*	Myrtaceae	Leaves	Pick fresh guava leaves, then grind them until they are very fine, mix with very little water, squeeze and filter it well to get fluid, and give it to the child. For under-five year children, give the amount of a teaspoon three times per day for three consecutive days.
2	Mlama – (Swahili/Zigua)	*Combretum molle*	Combretaceae	Roots	Dig fresh roots, wash and soak them for a few hours to get a liquid substance, and give it to the patient to drink. One small cup three times a day for three days. Alternatively, dig fresh roots, wash and mix them with two cups of water, boil the medicine until one and a half cups of water remain, and then give it to the patient. You give him/her one cup of medicine twice a day for three days.
				Leaves	Pick fresh leaves, grind them well, mix them with very little water, squeeze and filter them well to get their fluid, and give them to the child. The child should drink two tablespoons once per day for three days.
				Roots or Barks	Fresh barks or roots should be dried and ground into powder before being mixed into porridge or warm water and given to the patient. One tablespoon powder in the porridge. For three days, three times every day.
3	Mdaula – (Swahili/Zigua)	*Zanha africana*	Sapindaceae	Roots	Dig fresh roots, remove the outer layer, wash them, mix with one litre of water, and boil until you get half a litre. The child should take two tablespoons three times a day for three days. Alternatively, dig fresh roots, take the outer layer, wash and grind them, then dry under the sun. The powder can be used with porridge or warm water. One tablespoon of powder three times per day for three days.
4	Mtula – (Zigua/Sambaa) Ndulele – (Swahili)	*Solanum incanum*	Solanaceae	Roots	Dig fresh roots, add one litre of water, and boil until you get half a litre. For seven days, the child consumes half a cup three times a day.
5	Mtundwi – (Zigua)	*Ximenia americana*	Olacaceae	Leaves	Pick fresh leaves, dry, and grind them until they become powder. Store it in a dry place (where there is no humidity). The medicine can be used with tea or porridge. The child should take one teaspoon of powder three times per day until the child recovers.
				Roots	Dig the roots and wash them well, then mix with water and boil. If it is one litre of water, boil it until you get half a litre. The child takes a quarter of a cup, three times a day until the child recovers.
6	Mkumbi (Zigua/Swahili	*Ochna holstii*	Ochnaceae	Barks	Pick the barks from the tree, grind them well to get the powder, sift, and store the powder in a bottle. The medicine is used by mixing it with hot water half a litre with one tablespoon of medicine. The child should take two tablespoons three times a day for three or four days.
7	Mnenekanda (Zigua/Swahili)	*Elaeodendron schlechtarianum*	Celastraceae	Leaves	Pluck fresh leaves, grind them well, squeeze, and filter well to get the juice. Store it in a cool place. The child takes two teaspoons three times a day for two days.
				Barks	Take the barks, dry them before grinding them to get a powder, and then mix the powder with water and give one tablespoon to a child once per day until the child recovers.
8	Kivumbasi/Mzumbasha – (Sambaa/Zigua)	*Ocimum suave*	Lamiaceae	Leaves	Pluck fresh leaves, grind them well, mix with a litre of water, squeeze, and filter well to get a watery substance. The child takes the amount of liquid, three or four tablespoons, three times, a day for two days.
				Roots	Dig fresh roots, wash them well, and then mix with water and boil. If it is one litre of water, boil it until you get half a litre. The child should take half a cup three times a day for three days.
				Leaves and Roots	Take fresh leaves and roots, dry them properly, and grind to get a powder. Add a small amount of hot water and use it. The child takes one tablespoon once per day until s/he recovers. The dose of the medicine can be increased accordingly to a quarter of a cup, depending on the age of the child.
9	Chegonde - (Sambaa), (Mkumba in Swahili)	*Rhus natalensis*	Anacardiaceae	Leaves	Pick fresh leaves, grind them well, and soak for a quarter of an hour or half an hour. The child takes the amount of six tablespoons: two in the morning, two in the afternoon, and two in the evening for three days
10	Mlwati (Zigua)	*Dombeya rotundifolia*	Malvaceae	Leaves	Grind fresh leaves, squeeze, and filter them well to get a liquid substance. The child takes one tablespoon twice a day for one or two days.
				Cords or the Barks	Peal the cords or the barks and soak them in water for one hour. The child takes the amount of one cup or half of a cup twice a day for one or two days.
11	Mkwaju - (Swahili)	*Tamarindus indica*	Fabaceae	Leaves	Grind fresh leaves, mangle them, and filter well to get a liquid substance. The child takes the amount of one tablespoon twice or three times a day for two or three days.
12	Mkala – (Zigua)	*Ozoroa insignis*	Anacardiaceae	Leaves	Grind fresh leaves, squeeze, and filter them well to get the liquid substance. The child takes two teaspoons three times a day for two days.
13	Mfuleta – (Swahili)	*Albizia anthelmintica*	Fabaceae	Barks	Peel off the bark’s outer layer to remain with the cords. Mix the cords with one and a half cups of water and boil it a little. The child takes four tablespoons twice a day until the child recovers. Alternatively, peel the barks and soak them in water for one day only; the purpose is to get the sauce. The child takes the amount of a teaspoon twice a day until the child recovers.
14	Mkole - (Sambaa)	*Grewia sp*	Malvaceae	Leaves	Grind the leaves, and after that, boil and filter before you give it to the child to drink. The child takes two teaspoons once a day until the child recovers.
15	Mkusu - (Swahili)	*Harrisonia abyssinica*	Rutaceae	Leaves	Crush fresh, tender leaves; filter them well to get the liquid substance. The child takes two teaspoons twice a day for two or three days.
16	Mwembe - (Swahili)	*Mangifera indica*	Anacardiaceae	Leaves	Obtain fresh leaves, grind them well, mix with seven tablespoons of water, squeeze and filter well to get some sort of juice, and give it to the child. The child should drink three teaspoons every six hours for three days.
17	Mtwintwi – (Zigua)	*Commiphora pteleifolia*	Burseraceae	Barks	Peal the barks and soak them in the water for a few hours in order to get a liquid substance. The Child takes half a cup three times per day for five days.
				Leaves	Pick fresh leaves, grind and mix them with water, wait for half an hour, then squeeze and put the medicine in a clean bottle. The child takes two tablespoons three times per day for three days.
18	Mwengele/Muhengele - (Zigua)	*Cyphostemma njegerre*	Vitaceae	Roots	Dig the roots, prepare them well by cutting them into small pieces, soak the pieces in water, wait until you get some sort of thick liquid substance, and give it to the child. The child takes a small amount of medicine three times a day until s/he recovers. On average for 7 days.
19	Mlama Mweusi - (Swahili) Malamla (Zigua)	*Combretum molle*	Combretaceae	Roots	Dig fresh roots, wash them well, soak for a few hours, and give it to the patient to drink. The child takes the amount of one cup three times a day until s/he recovers.
20	Mharashambuzi - (Zigua)	*Indigofera swaziensis*	Fabaceae	Roots	Dig fresh roots, wash them well, soak for a few hours, and then add a little amount of sugar or honey; it treats diarrhoea and stimulates blood production as well. The child takes two tablespoons three times a day until s/he recovers.
21	Mumbu - (Zigua)	*Lannea schweinfurthii var. stuhlmannii*	Anacardiaceae	Barks	Obtain the outer layer, soak it for a few hours, and give it to the patient to drink. The child takes the amount of one teaspoon three times a day until s/he recovers.
22	Mkundekunde – (Swahili)	*Cassia abbreviata Oliv.*	Fabaceae	Roots	Dig fresh roots, wash them well, mix with some water, and boil well. If it is one litre of water, boil it until you get half a litre. The child takes half a cup three times per day for up to three days.
23	Mnama – (Pare)	*Combretum molle*	Combretaceae	Barks	Get the barks, wash them well, strain, dry, and grind. Take the powder obtained from the barks and store it in a good place; wherever you store it, it must be clean. Then you take clean, boiled, and boiled water and put it in a cup, and the child may drink it. The child takes the amount of four tablespoons three times per day until s/he recovers. At this time, the child should also be drinking a lot of water, and s/he can take a mixture of oranges, lemons, and a little sugar.
24	Mkongowe – (Zigua)	*Acacia gerrardii*	Fabaceae	Roots	Dig fresh roots, wash them well, mix with some water, and soak for a few hours to get the fluid. The child takes one tablespoon every day for a week.
25	Leza - (Sambaa)	*Ehretia sylvatica*	Boraginaceae	Leaves	Pick fresh leaves, grind them, and mix with milk. If you don’t have milk, you can prepare by soaking them in water for some time, and then give the liquid substance to the patient. The child takes the amount of a tablespoon and takes it in the morning, then by noon s/he feels better.
26	Mshegheshe-(Sambaa)	*Myrica salicifolia*	Myricaceae	Leaves	Get fresh leaves, grind and mix them with a little water, then squeeze and filter to get a liquid substance. The child takes the amount of one tablespoon three times per day until s/he recovers.
27	Mhafie - (Sambaa)	*Millettia lasiantha*	Fabaceae	Barks	Peel the barks, grind them, and let them dry. When dry, filter, mix with hot water, and then give it to the patient according to his or her age. Under-five children are required to take one teaspoon of medication for two days only, and diarrhoea will stop completely.
28	Mtusi - (Zigua)	*Vachellia nilotica*	Fabaceae	Barks	Peel the barks and soak them for a few hours, then squeeze and filter to get a liquid substance. The child can take a small amount of medicine, one tablespoon twice daily, until s/he recovers.
29	Mgunku - (Zigua)	*Stereospermum kunthianum*	Bignoniaceae	Barks	Take the barks and soak them in water for a few hours, then squeeze and filter to get a liquid substance. The child can take a small amount of medicine—one tablespoon twice daily—until s/he recovers, usually for three days.
30	Makandiza - (Zigua)	*Psorospermum febrifugum*	Clusiaceae	Leaves	Get fresh leaves, grind and mix them with a little water, then squeeze and filter to get a liquid substance. The child takes the amount of two or three tablespoons once per day for two or three days, depending on the progress of the patient.
31	Mtulavuha - (Zigua)	*Rytigynia schumannii*	Rubiaceae	Roots	Dig fresh roots, wash them, mix with some water, and boil the roots well. If it is one litre of water, boil it until you get half a litre. The child takes two tablespoons three times per day. The patient’s progress will determine the number of days s/he will take the medicine.
32	Mkatakwa - (Zigua)	*Brackenridgea zanguebarica*	Ochnaceae	Barks	Get fresh barks from the tree and dry them. Not the inner barks, but the outer barks. Grind them to get the powder. Then boil water and put about one tablespoon of herbal powder in the water. Mix it well and then give it to the child, in the amount of one tablespoon of medicine, three times a day for three to four days.
33	Mnamang’ombe - (Zigua)	*Combretum collinum*	Combretaceae	Barks	Get the barks from the tree. Take the barks from the side of the sunrise. When harvesting, the barks that will be facing up by the time they are falling down from the tree are the ones that are supposed to be taken; however, those tree barks that have fallen and look down are not appropriate and should be abandoned. After getting the relevant barks, soak them in water for a few hours, then squeeze and filter to get a liquid substance. The child takes the amount of one teaspoon three times per day until s/he recovers.
34	Hoza - (Zigua and Swahili)	*Cissus aralioides*	Vitaceae	Leaves	Get fresh leaves and grind them until they become slimy. Stir in water and give it to a patient. The child takes one tablespoon twice per day for four days.
35	Konko - (Zigua)	*Polyscias stuhlmannii*	Araliaceae	Leaves	Get fresh leaves, grind and mix them with half a cup of water, then squeeze and filter to get a liquid substance. The liquid is administered to the child. Half a cup is divided into two, and the child only drinks it twice a day, in the morning and in the evening.
36	Mkwizinge - (Swahili)	*Cassia abbreviata*	Fabaceae	Roots	Dig fresh roots, wash them, mix with some water, and boil the roots well. If it is one litre of water, boil it until you get half a litre. The child takes half a cup three times per day for seven days.
37	Mvungaliza - (Zigua)	*Synaptolepis kirkii*	Thymelaeaceae	Leaves	Get fresh leaves of Mvungaliza (*Synaptolepis kirkii)*, Mtwintwi *(Commiphora pteleifolia)*, and Guava leaves, grind them together until it becomes slimy, and add water. With the water in the mill, keep on grinding as if to make a stew. After this, filter the water, and the medicine can be administered to the child according to their age. A quarter cup, half a cup, or a whole cup, according to their age. If it’s a 4- or 5-year-old, they can take a whole cup. If they are younger, say 2- or 3-year-old, you give them half a cup once per day until s/he recovers.
38	Kongukubwa - (Zigua)	*Inhambanella henriquezii*	Sapotaceae	Leaves	Pick fresh leaves, grind them well, and soak in water for five to ten minutes. Squeeze and filter it well to get the liquid substance. The child takes a quarter of a cup in the morning and evening for five days.
39	Mkongodeka - (Zigua)	*Grewia microcarpa*	Malvaceae	Leaves	Pick fresh leaves of Mkongodeka *(Grewia microcarpa)* and Guava leaves, grind them together well, and let it dry. The ground leaves need to dry well in a shady place because, when exposed to the sun for long, powder loses its healing properties. After drying, the medicine can be administered to the child by adding it to porridge or hot water. The child takes one tablespoon three times per day for three or four days.
40	Mhombe - (Zigua)	*Heeria* *reticulata*	Anacardiaceae	Roots	Fresh roots should be dug, washed, peeled, and dried. Thereafter, grind them until are powdery. The child is required to take the amount of one tablespoon of powder mixed with a glass of water three times per day for the period of one week.
41	Tungotungo - (Zigua)	*Strychnos innocua*	Loganiaceae	Roots	Dig fresh roots, peel them, and cut into pieces. Put in the sun to dry. Then grind until they form powder. The child is required to take one tablespoon of powder mixed with a glass of water three times per day for seven days, and s/he should not stop taking medicine even if s/he gets better.
42	Mvungawiza - (Sambaa)	*Xymalos monospora*	Monimiaceae	Roots	Dig fresh roots, wash and dry them, and then grind until they become powder. Take a teaspoon of this medicine and soak it in a cup of water for half an hour. Give the liquid substance to the patient to drink. If it is a young child, give him or her one teaspoon twice a day (morning and evening) for four days.
43	Mgotu - (Zigua)	*Diospyros fischeri*	Ebenaceae	Roots	Dig up fresh roots and cut them into pieces. Soak in water for a few hours to get the liquid substance and give it to the child to drink. The child takes one cup of medicine three times per day for four days.
44	Nyejeza - (Zigua)	*Kigelia africana*	Bignoniacaea	Leaves	Get fresh leaves, grind them, then squeeze and filter to get the juice. The liquid substance obtained is administered to the child. The child takes the amount of one tablespoon of medicine only once, and after a few minutes, s/he will feel better.
45	Mnukapala - (Sambaa)	*Zanthoxylum chalybeum*	Rutaceae	Leaves	Pick fresh leaves, grind them, and dry in the sun, and then mix the herbal powder (less than a tablespoon of herbal powder) with a quarter cup of water and give it to the child twice a day, or if he has nonstop diarrhoea, s/he can take it three times a day until s/he recovers.
46	Mchimbu - (Zigua)	*Dombeya rotundifolia*	Sterculiaceae	Roots	Dig the roots and wash them well, then mix with water and boil. If it is one litre of water, boil it until you get half a litre. The child takes the amount of a quarter of a cup because a child might spill some when they get medication. They can be given three times per day for three days.
47	Mnungumagoma - (Zigua/Swahili)	*Erythrina abyssinica*	Fabaceae	Roots	Dig fresh roots, wash them, soak in water for a few hours to get the liquid substance, and then give it to the child. The child takes the amount of one tablespoon three times per day for only one day.
48	Kiandama - (Sambaa)	*Asparagus flagellaris*	Asparagaceae	Leaves	Pick fresh leaves, grind them, and mix with water. Then squeeze and filter to get a watery substance. The child takes the amount of a teaspoon in the morning, afternoon, and evening for three days.
49	Mzuma - (Sambaa)	*Harrisonia abyssinica*	Rutaceae	Leaves	Pick fresh leaves, grind them, and mix with water. Then squeeze and filter to get a watery substance. The child takes the amount of a teaspoon in the morning, afternoon, and evening for three days.
50	Mzindanguwe - (Sambaa)	*Blighia unijugata*	Sapindaceae	Leaves	Pick fresh leaves, wash them, grind, mix with water, and filter before you give it to the child or an adult to drink. The child takes three teaspoons in the morning, afternoon, and evening for only one day.
51	Sutang’andu - (Zigua)	*Salacia stuhlmanniana*	Celastraceaea	Leaves	Pick fresh leaves, grind and mix them with a little water, and then squeeze to get a liquid substance. The child takes two tablespoons three times per day for three days.
52	Mfundofundo (Sambaa) – (Swahili - Kifundo)	*Zenkerella grotei*	Fabaceae	Leaves	Pick fresh leaves, grind and mix them with a little water, and then squeeze to get a liquid substance. The child takes a tablespoon three times per day for three days. An adult can chew these leaves, swallow them, and drink some water.
53	Mhaikanyoya - (Zigua)	*Deinbollia borbonica*	Sapindaceae	Roots	Dig fresh roots of Mhaikanyoya (*Deinbollia borbonica*) and Mtura (*Solanum incanum*), dice them, and boil together. If it is two litre of water, then boil it until you get one litre. If the child is under five years old but only one year old, then s/he’ll drink a quarter of a cup of tea. If s/he’s about 4 years old, then s/he can drink half a cup, and the 5-year-old can drink even a whole cup of tea, three times per day for three to seven days, depending on the condition of the patient.
54	Sitahula – Sambaa (Mzangaze - Swahili)	*Achyranthes aspera*	Amaranthaceae	Leaves	Pick fresh leaves, crush and combine them with two litres of water, then squeeze and filter to obtain the liquid ingredient. The child must take one cup of medication three times every day until s/he recovers. The medication causes the stool to harden fast.

### Therapeutic effects of herbal medicines relevant for the treatment of diarrhoeal Diseases

All interviewees thought that herbal medicines had enormous and unmistakable therapeutic effects in the treatment of diarrhoeal diseases among under-five children. Participants emphasized that herbal medications are an essential intervention in the battle against diarrhoeal illnesses. It was the majority of participants’ dream and wishes to see the world’s treasure of herbal remedies used to benefit and aid the needy:

*“I am quite sure these medicines have the great potential to treat diarrhoea in children”* Participant no 24, Male, 32-year-old.

*“They are definitely capable of curing diarrhoeal diseases”* Participant no 12, Female, 72-year-old.

*“It stops and heals diarrhoea completely. Many children who are brought here, the next morning, the parents would tell you, Mum, my child is doing well”* Participant no 24, Female, 32-year-old.

*“Many people are drawn to herbal medicines because they heal quickly and have no side effects”* Participant no 31, Male 42-year-old.

*“You discover that the child has had diarrhoea for a long time, and the caretaker purchased pills and gave them to the child without success, and s/he knows that if she takes a certain herb and gives it to the child, s/he will recover”* Participant no 43, Female 67-year-old.

*“Herbal medicines have the potential to treat diarrhoea, even chronic diarrhoea.“ We normally blend more than one medicinal plant to produce one remedy”* Participant no 12, Male, 66-year-old.

On combining more than one medicinal plant, participants narrated:

*“You can mix two herbal medicines, Mdaula (Zanha africana -* Sapindaceae family*) and Mtundwi (Ximenia americana –* Olacaceae family*). For mdaula (Zanha africana -* Sapindaceae family*), you dig the roots, remove the outer layer, and mix with the leaves of mtundwi (Ximenia americana –* Olacaceae family*). You dry them and grind until they are powdery, then store in a good place where there is no humidity. For me, usually, for this medication, a person cannot come right away and get it. I have to prepare it beforehand. The child takes three tablespoons three times a day: in the morning, one tablespoonful, in the afternoon, and in the evening. You can mix it with the porridge or even tea, but not milk, because milk attenuates it”* Participant no 19, Male, 52-year-old.

*“There is a medicinal plant called Mhaikanyoya (Deinbollia borbonica –* Sapindaceae family*). For this one, you dig out the roots and mix them with the roots of “Ndulele”, We call it “Mtula (Solanum incanum -* Solanaceae family*). Even if the child has bloody diarrhoea or if it’s just ordinary food diarrhoea mixed with blood, it will be treated. It will stop the diarrhoea immediately. For a child aged five years and below, after you’ve dug out the roots, diced them, and boil on a stove, depending on whether or not the child is under five years old but only one year old, s/he’ll drink quarter a cup. If s/he’s about four years old, s/he can drink half a cup, and a 5-year-old can drink even the whole cup of liquid herbal substance. It will stop the diarrhoea immediately”* Participant no 11, Male, 65-year-old.

When asked about the dosage, the participant had this to say

*“You’ll instruct them to take it for three days, but they usually don’t take that long. After taking it three times a day, you won’t see diarrhoea by the second day. But you tell them to give it to the child for seven days, morning, afternoon, and evening, but, sir, it does not take them that long”* Participant no 11, Male, 65-year-old.

Other participants went even further, claiming that herbal treatments may occasionally heal diarrhoea more effectively than medications prescribed in hospitals. Some participants were of the view that there are caretakers who take their children to the hospital for diarrhoeal treatment, but they do not recover. However, if they use herbal medicines, they recover completely.

*“Yes, the diarrhoea will stop.“ I’ve worked with children as well as adults. I know that occasionally they’ll inform me that their children have finished the hospital medication but still have diarrhoea. “I’m confident that if they use my remedy, their diarrhoea will stop”* Participant no 37, Female, 50-year-old.

*“These medicines have the ability to treat very well, sometimes better than hospital medicines”* Participant no 08, Male, 51- year-old.

*“Herbal medicines can completely cure diarrhoea without requiring the patient to visit a hospital.“ They shouldn’t”* Participant no 08, Male, 65-year-old.

Furthermore, participants stated that a substantial number of individuals, particularly in remote settlements and nowadays even in urban areas, rely exclusively on their services. When asked why the majority of people mostly prefer to use herbal medicines, they articulated that people have belief, faith, and trust in herbal medicines that are capable of treating diarrhoeal diseases among children. For example, participants had this to say:

*“The main reason is their belief in herbal medicines because these remedies have the great potential to treat faster than tablets”* IDI, Participant No 51, Male, 54-year-old.

*“The biggest reason is the belief of being told that this is the right medicine; when you take it, you will be healed, and probably others have used it and were cured, so that would motivate them to use the medicine”* IDI, Participant No 17, Male, 42-year-old.

*“They believe that herbal medicines are more effective in treating diarrhoea,”* IDI, Participant No 33, Female, 72-year-old.

It was reported that there is a special plant for treating diarrhoea that is caused by superstitious beliefs, also called *“kupigwa zongo”* in the local language (Bewitchment by zongo). According to participants, this form of diarrhoea cannot be cured with modern medication, leaving only herbal medicine as a therapy option.

*“And if s/he has diarrhoea because s/he has been bewitched by “Zongo”, you give him/her herbal medicines called Ndulele/Mtula (Solanum incanum plant*, Solanaceae family*)”* Participant no 09, Male, 64-year-old.

*“You know diseases are different; if someone is bewitched by Zongo,“ the hospital tests cannot detect the problem, but when s/he comes to us, we can see it.“* Participant no 35, Female, 35-year-old.

*“They use herbal medicines because they know that there are various diseases that hospital medications cannot cure, but herbal medicines can; for example, “Zongo”* IDI, Participant No 03, Female, 51-year-old.

*“It is true that “Zongo” causes a child to have diarrhoea, and if it is not treated with herbal medicines, no relief will be found even if you take him/her to the hospital”* Participant no 24, Male, 32-year-old.

*“They often use herbal medicines because they think the child has been bewitched by Zongo”* Participant no 41, Female, 37-year-old.

Participants suggested that diarrhoea due to superstition *(Zongo)* is a common belief that has been built among many residents living in the area where the study was conducted:

*“Many people use herbal medicines because they believe in them, and especially in our Region, Tanga, there is a problem of “Zongo” to a large extent, so once a child has diarrhoea, the parent associates it with “Zongo” and s/he believes that herbal medicines are the ones that can treat it”* Participant no 51, Male, 54-year-old.

A minority of participants, on the other hand, believed that plant-based medications are just for first aid and not for full treatment. As a result, they believed that even after being treated with herbal medications, it is still necessary to visit a health centre for continued clinical treatment. Participants were of the opinion that attending to the hospital would result in additional tests and medical advice, which would improve the patient’s care:

*“Although my advice is that after taking herbal medication, they should also go to the hospital to get checked and try to find out why diarrhoea started in the first place, as it can be caused by many things”* Participant no 10, Male, 66-year-old.

*“I should say that the use of herbal medicines is like first aid, and if the first aid fails, then the patient will have to go to the Hospital for complete treatment”* Participant no 07, Male, 59-year-old.

## Discussion

This research has explored the medicinal plants used by study participants to treat diarrhoeal diseases among under-five children in Korogwe and Handeni districts, Tanzania. According to the study findings, all interviewed participants had treated under-five children with diarrhoea with herbal medications on many occasions, which is consistent with earlier research carried out in Africa [12, 13]. Traditional healers were of the views that their competence in treating various ailments, including diarrhoea, is the cornerstone of the community’s faith in them. This attitude could be attributed to a lack of exposure to the quality and effectiveness of modern medicines, which are thought to be therapeutically superior to herbal medicines in treating various illnesses for the reason that they have undergone various stages of research and have been approved for human use [32]. Traditional healers must understand that prescribing untested and unlicensed herbs to patients is illegal and might cause more damage than good. Relevant authorities should invest in educating THs and the general public, particularly in rural areas, about the possible negative effects of taking untested herbal remedies. Consistent civic health education, particularly in rural settings, can aid the community better grasp a variety of health-related topics [33, 34].

According to studies, relevant medical formal education is the most basic necessity for health-care workers. As a result, everyone who offers health services to humans or any living creature must have a suitable certification that allows him or her to serve people or living animals [34, 35]. In contrast, the findings of this study suggest that all THs have never had any formal training related to herbal medicines, yet they continue to offer treatment to individuals like other health professionals, which is unsafe and can damage the health of herbal medicine consumers. Furthermore, the findings suggest that the majority of THs learned their skills from their forefathers (parents or guardians) or grandparents. A research conducted in Uganda showed a similar outcome [4]. This is erroneous since anyone who is not medically trained and/or certified by the relevant authorities is legally prohibited from treating any living species. Therefore, government authorities should impose a limit on the number of THs who are not licensed and/or qualified to continue treating patients, and they should promote and support them in obtaining the necessary training and certifications.

A total of 54 medicinal plants have been reported by the participants to have the required effectiveness in curing diarrhoeal diseases among under-five children (Table 2). Out of 54 medicinal plants, 15 plants were largely reported by the majority of participants. Those medicinal plants include *Psidium guajava* (Myrtaceae), *Rhus natalensis*, (Anacardiaceae) *Ozoroa insignis* (Anacardiaceae), *Tamarindus indica* (Fabaceae), *Ocimum suave* (Lamiaceae), *Combretum molle* (Combretaceae), *Zanha africana* (Sapindaceae), *Solanum incanum* (Solanaceae) and *Ximenia americana* (Olacaceae). Other medicinal plants mentioned by most participants include, *Ochna holstii* (Ochnaceae), *Elaeodendron schlechterianum* (Celastraceae), *Albizia anthelmintica* (Fabaceae), *Commiphora pteleifolia* (Burseraceae), *Salacia stuhlmanniana* (Celastraceae) and *Zenkerella grotei* (Fabaceae). The medicinal plants identified by Tanzanian THs have also been reported by other studies to be effective in healing diarrhoeal disorders [12, 13, 20]. Having THs who are well versed with medicinal plants is promising for the country, and it is vital that the government authorities see how they can seize this important knowledge and use it for the present and next generations. In addition, the findings from this study should encourage systematic botanical research to find out whether or not the herbal medicines reported in the present study have anti-diarrhoeal properties. Should these remedies be proven to have anti-diarrhoeal properties, then relevant agencies should consider recommending that they go through clinical trial processes and finally be approved for human use.

All of the therapeutic plants reported by participants were found in the environment where people inhabit. Traditional healers often select the right medicinal plants, pick the right parts of the plant, and prepare, store, or prescribe them right away. A similar finding was also reported in a different study carried out in Eastern Tanzania [9]. The methods for preparing herbs were totally dependent on the instruction provided to the TH in question. Although these herbs have been extensively used to heal under-five children, the unexpected thing is that none of the questioned TH has ever registered his/her herbs, making the use of their treatments arbitrary, which, in the end, may cause harm to the users. Tanzania’s government, through the Ministry of Health (MoH), has established processes for recognizing and registering THs and their herbal remedies once they have been certified for human use by the appropriate local authorities [15]. Therefore, deliberate efforts should be made by relevant government agencies across the country in partnership with THs themselves to ensure that all THs are adhering by this regulation in order to make their practice and products legally and therapeutically suitable for human use.

Based on TH’s responses, the roots, leaves, and barks of the respective medicinal plants were the parts mostly reported as the main source of medicine (Table 2). This result is in line with studies conducted in South Africa, Palestine, and Tanzania [6, 11, 12]. In general, the preparation of herbal medicine by using leaves or roots is done by picking or digging fresh leaves/roots, grinding them well, and then mixing with a little amount of water, squeezing, and filtering well to get the watery substance (juice). After that, the herbal remedy is ready for use. Alternatively, the medicine is prepared by taking the leaves, barks or roots, any of these, then cutting them into small pieces, let them to dry in the sun or shade, and then grinding them well until they become powder. After finishing these steps, the herbal remedy is ready for use. In addition, the medicine can be made by taking the roots or barks, cutting them into small pieces, and soaking in water for some time to get the liquid substance. After that, the herbal remedy is ready for use. The dosage of the medicine varies depending on the age and condition of the child. Although THs demonstrated that they had the knowledge necessary to prepare and prescribe herbal medications, more training is still necessary to ensure that their services are up to standards. As opposed to the current situation, where you might find that although some THs have comparable preparation and prescription processes, others have different processes.

As for the therapeutic potential of herbal medicines, the findings demonstrated that the remedies have a great ability to treat diarrhoeal diseases among under-five children. Similar findings were reported in other studies conducted in Tanzania, Ghana, and Ethiopia, where herbal medicines were found to have efficacy to treat diarrhoeal diseases in both adults and children [11, 20, 21]. The study participants appeared to have a high level of trust in herbal medicines for treating diarrhoeal ailments, even indicating that herbal medicines are superior to contemporary treatments for treating a variety of conditions, including diarrhoeal diseases. Furthermore, a few participants dared to say that they occasionally encourage their patients not to go to the hospital after being treated with herbal medications. This confidence on the perceived efficacy of herbal medicines is unsafe and may cause more health problems among under-five children given that most of herbal medicines, despite their claimed therapeutic potential, they lack quality and standard [5, 6, 21]. More in-depth studies are needed to determine if herbal remedies have powerful chemical compounds that can combat diarrhoeal pathogens.

Diarrhoea among under-five children is very common in both districts, Korogwe and Handeni [22]. Similarly, the use of herbal remedies in the treatment of diarrhoeal disorders is widespread across Tanga Region [24]. Usually, the decision of which diarrhoea medicine the patient should be given depends on the condition and age of the patient after the TH has taken an illness history. For normal diarrhoea, there are various herbal medicines that are claimed to have efficacy capable of treating this form of diarrhoea, depending on the understanding of a specific TH. However, for diarrhoea that is thought to be caused by superstitions, there is a unique treatment for this type of diarrhoea. The study findings suggest that there are still THs who attribute diarrhoeal diseases to mystical causes and fancy treatment by using herbal medicines rather than going to the health facility. Interestingly, a large number of THs believe this matter is real, which is in line with a number of studies conducted in Africa and other countries [36–38]. Such THs’ views may have been due to the training that was given to them by their elders, and maybe in reality there is no truth because even when THs were given the opportunity to explain how magic can cause diarrhoea, they could not give clear answers. Therefore, to handle this attitude, THs should be encouraged to take time to learn more about the biomedical causes of diarrhoeal diseases and other ailments with support from the relevant government authorities. Keeping on learning will help THs supplement their understanding of issues related to their undertakings of treating patients by using herbal medicines.

On the other hand, the findings show that there are a few THs who believe that caretakers will still have to take their under-five-year-old children to the hospital for further medical examinations even though their remedies have already cured the children, or vice versa, which is similar to a finding by *Welz et al.*, [39]. The practice of advising caretakers to seek additional medical care from the health facility is impressive given that THs do not have diagnostic tests that can ascertain the source and cause of the illness, even if the patient has recovered. Therefore, there is a need for herbal medicine policymakers to reach out to THs and equip them with more knowledge on the importance of advising their patients to seek additional medical care from modern health facilities even if they have already treated them.

### Lesson Learnt

The study findings imply that THs had a vast knowledge of medicinal plants used for treatment of diarrhoeal diseases among under-five children. Therefore, since medicinal plant knowledge is mostly passed down through families, there is a need for relevant stakeholders to figure out how to grasp this key knowledge, which is not taught in formal education systems, so that it can be a source of information for current and future generations. At the moment, it appears that knowledge of medicinal plants is possessed informally by a few people, particularly THs, which is a potential risk. The understanding of medicinal plants is also critical to the overall conservation plans of natural forests, which are a reliable resource of medicinal plants.

Regarding safety and efficacy, some herbal medicines have been described as safe and highly effective in treating diarrhoeal diseases. In order to substantiate this conviction scientifically, more thorough research is needed to investigate whether herbal medicines really have therapeutic effects that can fight diarrhoeal pathogens.

## Conclusion

A total of 54 medicinal plants have been reported to have therapeutic benefits for healing diarrhoeal diseases among under-five children. Nonetheless, out of 54 medicinal plants, 15 were mostly revealed by the majority of participants. All THs acknowledged that they have been treating under-five children with diarrhoea by using herbal medicines on multiple occasions. Although the use of these herbs seems to be flourishing in the study setting, the authorities must monitor their use since, aside from their adoption; these herbs have never been thoroughly researched to prove their safety and efficacy. It should be understood that the arbitrary use of untested and unapproved herbal medications might have negative consequences for their users. According to studies, there are herbal medications that have been adequately researched and confirmed to be beneficial in treating numerous ailments, including diarrhoea among under-five children, even so there are also herbal remedies that have been studied and found to be harmful. Therefore, a rigorous botanical study will shed more light on the usefulness and limitations of these herbal medications.

### Recommendations

A more complete investigation on the safety and effectiveness of herbal medicines is necessary to determine if herbal remedies have therapeutic advantages that can combat diarrhoeal illnesses. Additionally, given that registration is required by law, those THs who haven’t done so should think about registering both themselves and their herbs. Giving patients herbs that have not been approved by competent authorities and providing health services without a license is dangerous for the users and can have major negative health consequences. Furthermore, THs should refrain from persuading patients not to visit the health facility before or after receiving their treatment, and they should stop exaggerating the benefits of herbal remedies as a form of therapy. Traditional healers should always advise their clients and patients to visit the medical facility and get tested before they begin seeing them so that the health problem is clinically diagnosed rather than speculating as to the patient’s illness and ultimately failing to provide the patient with the care s/he deserves.

## Data Availability

The dataset generated and/or analyzed in this study will be available from the corresponding author on reasonable request with permission from the University of Dodoma.

## List of abbreviations

BNITM: Bernhard Nocht Institute for Tropical Medicine
ICF: Informed Consent Form
IDIs: In-depth Interviews
MoH: Ministry of Health
NIMR: National Institute for Medical Research
PI: Principal Investigator.
TDHS: Tanzania Demographic and Health Survey
THs: Traditional Healer/s
UNICEF: United Nations Children’s Fund
UDOM: University of Dodoma
IRRC: Institutional Research Review Committee
WHO: World Health Organization
